# Beyond the screen: Lived experiences and coping strategies of educators facing digital eye strain in a Philippine university

**DOI:** 10.1016/j.qrmh.2026.100046

**Published:** 2026-03-12

**Authors:** Alex S. Borromeo

**Affiliations:** College of Nursing, Bulacan State University, Philippines

**Keywords:** Digital eye strain, University educators, Ergonomics, Work–life integration, Coping strategies, Technostress

## Abstract

**Purpose:**

Prolonged digital screen exposure among university educators has led to increased cases of digital eye strain (DES), a growing occupational health concern in the academic sector. This study explored the lived experiences and coping strategies of faculty members facing moderate-to-severe DES at a Philippine university.

**Methods:**

A descriptive phenomenological approach guided this qualitative inquiry. Nine faculty members were purposively selected based on moderate-to-severe DES scores from a prior university-wide quantitative survey. Semi-structured interviews were conducted, transcribed verbatim, and analyzed using phenomenological analysis following Creswell’s descriptive phenomenological steps, including identification of significant statements, formulation of meaning units, and synthesis of themes describing the essence of the experience.

**Results:**

The findings revealed four major themes: digital health and resilience, workstation and environmental ergonomics, work-life integration, and health services, policy supports, and system-level enablers. Participants described the paradox of digital tools that enhance teaching while contributing to physical strain and technostress.

**Conclusions:**

This study underscores the urgency for educational institutions to integrate ocular health and ergonomic practices into workplace policies. Targeted interventions, such as regulated screen breaks, eye care programs, and digital wellness education, are essential. The findings contribute to sustainable development goals (SDGs) and advocate for a safer, health-conscious academic environment.

## Introduction

The widespread adoption of digital technology has significantly transformed higher education, a shift that accelerated during and after the COVID-19 pandemic. With the rapid transition to online and hybrid teaching modalities, university educators have become increasingly reliant on digital platforms for instruction, research, assessment, and administrative work (Madheswaran et al., 2024). While this digital integration has enhanced accessibility and operational efficiency, it has also introduced emerging occupational health concerns that warrant closer attention.

One such concern is digital eye strain (DES), also referred to in clinical literature as "computer vision syndrome" (CVS) (Blehm et al., 2005, Meneses-Claudio et al., 2023). DES refers to a cluster of visual and physical symptoms associated with prolonged digital screen use, including blurred or double vision, ocular fatigue, dry or irritated eyes, headaches, and neck or shoulder discomfort (American Optometric Association, 2017). University educators are particularly vulnerable due to sustained screen engagement required for lesson preparation, virtual instruction, grading, research productivity, and administrative responsibilities, often extending beyond formal working hours. Common mitigation strategies for DES include ergonomic screen setup (appropriate viewing distance, screen height, posture, and lighting or glare control), scheduled visual breaks (e.g., the 20–20–20 rule), and preventive eye care such as vision screening and computer-optimized corrective lenses.

Although DES is primarily a vision-related condition, it occurs within digitally intensive work environments characterized by heightened psychosocial demands. In this context, technostress—defined as psychological strain arising from constant connectivity, information overload, rapid technological change, and blurred work–life boundaries— provides an important contextual lens (Marrinhas et al., 2023, Tarafdar et al., 2007, Yang et al., 2025). While DES and technostress are conceptually distinct, they often coexist in academic settings where digital tools simultaneously enhance productivity and intensify workload pressures. In the present study, technostress is treated not as a parallel outcome, but as a contextual condition shaping how educators experience, interpret, and cope with DES. Recent work also suggests that emerging tools such as generative AI may introduce additional techno-stressors for educators through rapid technology change and increased cognitive demands (Khlaif et al., 2025).

Research on DES among educators has been predominantly quantitative, emphasizing prevalence, symptom severity, and ergonomic risk factors (Kaur et al., 2022, Prasetya et al., 2024). What remains underexplored is how educators experience DES within real institutional conditions—how they interpret symptoms, negotiate digitally intensified academic demands, and sustain coping amid workload pressure and limited organizational support (Imran et al., 2025, Suyo-Vega et al., 2024).

To address the gap in qualitative research, the present study adopts a descriptive phenomenological approach to examine the lived experiences and coping strategies of university educators experiencing DES. Conceptually, the study was guided by Lazarus and Folkman’s (1984) transactional theory of stress and coping, which frames DES as a work-related stressor shaped by cognitive appraisals and coping resources. Tarafdar et al.’s (2007) technostress model situates DES within digitally intensified work conditions such as techno-overload (increased workload through technology) and techno-invasion (work intruding into personal time), which can constrain recovery.

## Literature review

### Digital eye strain among educators

A growing body of literature documents the high prevalence of DES among educators whose work requires sustained digital screen engagement. Across studies, approximately 50% to over 80% of teachers report moderate to severe DES symptoms, with prevalence varying by screen exposure duration, task demands, and ergonomic conditions (Gushgari et al., 2024, Kaur et al., 2022). In Peru, more than half of surveyed university educators reported symptoms consistent with computer vision syndrome (Meneses-Claudio et al., 2023), while similar patterns have been observed among teachers in diverse educational contexts (Estrada-Araoz et al., 2023, Shet Parkar et al., 2024).

Beyond physical discomfort, DES has meaningful implications for academic work. Educators experiencing persistent symptoms report reduced concentration, slower task completion, and difficulties sustaining instructional and assessment activities, thereby affecting both occupational health and educational quality (Gushgari et al., 2024). Prolonged exposure without adequate preventive measures may also contribute to progressive visual discomfort and increased reliance on corrective lenses over time (Kaur et al., 2022). Collectively, these findings underscore DES as a legitimate occupational health concern rather than a transient inconvenience of digital work.

Globally, DES has been documented across professions that rely heavily on screen-based tasks. The Vision Council (2016) estimated that approximately 65% of adults in the United States experience symptoms associated with routine digital device use. In educational settings, where prolonged screen exposure is integral to teaching, grading, and administrative responsibilities, these trends highlight the persistent and systemic nature of DES among educators.

### Screen ergonomics and preventive measures

Ergonomic optimization is widely regarded as a primary strategy for preventing or alleviating symptoms of DES. Evidence-based recommendations emphasize appropriate viewing distance, positioning screens slightly below eye level, maintaining neutral posture, ensuring adequate seating support, and optimizing ambient lighting to reduce glare (Munshi et al., 2017). Empirical studies demonstrate that inadequate ergonomic conditions and insufficient rest breaks are strongly associated with increased DES severity (Zayed et al., 2021).

Educational and behavioral interventions have also shown promise. Individuals with greater ergonomic awareness and access to appropriate resources report fewer and less severe symptoms (Kaur et al., 2022). Structured eye health education programs have been shown to improve preventive practices, suggesting that awareness can translate into sustained behavioral change when supported institutionally (Park & Ahn, 2022). However, faculty members—particularly in developing country contexts—often lack access to ergonomically appropriate workstations and rely on improvised setups that compromise visual comfort.

Preventive eye care further contributes to DES management. Routine vision screening and access to protective eyewear—such as computer glasses with anti-reflective coatings or prescriptions optimized for intermediate viewing distances—can support comfort during prolonged digital work (American Optometric Association, 2017, Kaur et al., 2022). These measures are intended to reduce visual load rather than serve as diagnostic tools or cures for DES, yet they remain inconsistently available in academic settings.

### Context: Philippine policy and institutional support

At the policy level, the Philippine government has established occupational health frameworks relevant to digitally intensive work. Republic Act No. 11058 mandates employer responsibility for mitigating occupational risks, including those associated with prolonged computer use (Respicio & Co., 2025). The Civil Service Commission (1992) promotes wellness breaks for public-sector employees, while the Commission on Higher Education encourages alignment with national occupational health standards.

Despite these frameworks, implementation within universities remains uneven. Studies and institutional reports indicate limited access to ergonomic assessments, structured screen breaks, and formal eye care provisions for educators. Scholars increasingly emphasize that sustainable DES prevention requires organizational and policy-level support rather than sole reliance on individual adaptation (Park & Ahn, 2022).

In summary, the literature characterizes DES as a multifaceted occupational health concern shaped by visual demands, ergonomic conditions, and institutional practices. While quantitative studies have documented prevalence and risk factors, qualitative insight into how educators experience and navigate DES within institutional contexts remains limited—particularly in Philippine higher education. The present study addresses this gap by providing an in-depth, experiential perspective on educators’ lived experiences and coping strategies.

## Materials and methods

### Study design

This study employed a descriptive phenomenological design following Creswell’s (2013) adaptation of Husserlian phenomenology (Creswell & Poth, 2018), which emphasizes describing the essence of a phenomenon through participants’ first-person accounts rather than theory generation or interpretation. In line with Husserl’s philosophy, this approach involves bracketing preconceived notions to focus on participants’ descriptions of their lived experiences. Rather than interpreting or theorizing beyond what participants shared, the study sought to accurately capture how DES is perceived, felt, and managed by university educators. This design was well suited to the study objectives, as it enabled an in-depth exploration of the subjective impacts of prolonged digital screen use on educators’ professional and personal lives. Using Creswell’s descriptive phenomenological method, analysis involved identifying significant statements, formulating meaning units, and synthesizing themes that reflected the core elements of the experience.

### Participants and setting

This study was conducted at a public state university in the Philippines. Purposive sampling—a non-probability technique commonly used to identify information-rich cases relevant to a phenomenon of interest—was employed (Creswell, 2013, Patton, 2015). Faculty members who reported moderate-to-severe DES in a prior university-wide quantitative survey conducted by the research team were recruited. This survey assessed DES symptoms using standardized self-report measures and served as the sampling frame for the present qualitative phase. Participants were intentionally selected to ensure that all interviewees had direct, experience of the phenomenon under investigation.

The inclusion criteria were (a) current faculty member at the university (full-time or part-time); (b) 18 years of age or older; (c) self-reported moderate-to-severe DES symptoms (e.g., frequent headaches, eye pain, visual fatigue) based on the prior survey or an initial screening; and (d) willingness to participate in an in-depth interview. Efforts were made to include participants with diverse characteristics in terms of age, gender, academic rank, and teaching discipline. In total, nine faculty members (six females and three males) participated, with ages ranging from the mid-20s to over 60 years. Two senior professors aged 60 years and above were included to ensure generational breadth, as older educators may experience distinct challenges such as pre-existing vision conditions or lower digital fluency.

All participants provided written informed consent and were assured of confidentiality through the use of coded identifiers (e.g., P1, P2). Data collection occurred in 2023, during a period when the university had resumed in-person classes while maintaining substantial online instructional, administrative, and preparatory work. This hybrid teaching context resulted in sustained and often extended digital screen exposure among faculty members.

### Data collection

Data were collected through individual, in-depth semi-structured interviews designed to explore educators’ experiences with DES. An interview guide was developed to ensure consistency while allowing participants to narrate their experiences in their own terms. The guide covered five core areas: (1) screen usage and daily routines; (2) experiences with DES symptoms; (3) coping strategies and institutional support; (4) impact on professional functioning and ergonomics; and (5) future outlook and recommendations.

Drawing on Lazarus and Folkman’s (1984) transactional theory of stress and coping, questions were mapped to stressors, appraisal processes, coping responses, and available resources. Tarafdar et al.’s (2007) technostress model further informed probes related to techno-overload and techno-invasion (e.g., after-hours work and constant connectivity), providing contextual depth to participants’ DES experiences.

Participants were asked questions such as, “Can you describe your daily routine involving digital screen use?” and “How many hours do you typically spend on screens, and for what tasks?” Questions related to DES symptoms included prompts such as, “What symptoms have you personally experienced?” and “How do these symptoms affect your work and home life?” Coping strategies were explored through questions including, “What adjustments or supports have you used to manage DES, and how effective have these been?” Additional questions addressed productivity, job satisfaction, ergonomic practices, and suggestions for personal or institutional improvements.

Follow-up probes (e.g., “Can you describe a particularly difficult day with eye strain and how you coped?”) were used to elicit deeper reflection. Interviews were conducted face-to-face in locations selected by participants for comfort and privacy, including faculty offices, vacant classrooms, and quiet campus meeting rooms. Sessions lasted approximately 30–45 minutes and were scheduled at times convenient to participants to minimize fatigue. Interviews were conducted primarily in English, with occasional Filipino expressions; all Filipino segments were translated into English during transcription to preserve meaning and context.

With participants’ consent, interviews were audio-recorded, and field notes were taken during and immediately after each session to capture contextual details and non-verbal cues. Participants were informed that they could pause or terminate the interview at any time. When emotional discomfort arose, interviews were paused and resumed only when participants indicated readiness to continue.

### Data analysis

Qualitative data analysis followed Creswell’s descriptive phenomenological method and involved several iterative steps. First, audio-recorded interviews were transcribed verbatim. Each transcript was read multiple times to achieve immersion in the data. Initial coding was conducted across all transcripts at the meaning-unit level, with codes assigned to distinct ideas or experiences expressed by participants. Only after this dataset-wide coding step were codes grouped into categories and synthesized into themes through iterative comparison across transcripts.

Next, horizontalization was performed, wherein significant statements were identified across transcripts. In this study, significant statements refer to verbatim excerpts from interview transcripts that directly describe participants’ experiences, perceptions, emotions, or coping behaviors related to DES. These statements included descriptions of symptoms, workplace adjustments, emotional reactions, and perceived institutional support. Statements unrelated to DES or digital work were excluded at this stage. This approach aligns with Creswell’s phenomenological analytic procedures, which prioritize participants’ own words as the foundation for meaning making.

Using phenomenological reduction, overlapping or redundant statements were removed, and remaining statements were clustered into meaning units. These codes were then compared across transcripts and clustered into conceptually related categories. Through iterative comparison and synthesis, these categories were refined into overarching themes that captured shared patterns across participants’ experiences. Throughout analysis, bracketing was maintained via reflective journaling to minimize researcher bias.

Four major themes emerged, each encompassing several sub-themes. Textural descriptions (what participants experienced) and structural descriptions (how experiences occurred within context) were developed and integrated into a composite description representing the essence of living with DES as a university educator. For example, multiple statements describing eye pain, blurred vision, and headaches during prolonged grading and lesson preparation—followed by accounts of extending work beyond regular hours due to reduced productivity—were clustered into related meaning units and synthesized into the broader theme of work-life integration, reflecting how DES symptoms and digital workload jointly shaped participants’ daily functioning

To enhance credibility, member checking and an audit trail were employed. Participants reviewed summary interpretations to confirm resonance with their experiences, and analytic decisions were systematically documented to support transparency and rigor.

### Ethical considerations

This study was conducted in accordance with the ethical principles outlined in the Declaration of Helsinki and was approved by the university’s Institutional Research Ethics Committee. Prior to participation, all participants received detailed information about the study's purpose, procedures, and their rights, and provided written informed consent.

Participation in the study was voluntary. Participants were informed that they could withdraw at any point or decline to answer any question without penalty. To ensure confidentiality, personal identifiers were removed or coded (e.g., P1 and P2). Data were stored securely on a password-protected computer that was accessible only to the research team, and audio files were deleted after transcription to prevent any potential breach of privacy. Recruitment was conducted via email to avoid coercion, and interviews were scheduled at participants’ convenience, in safe and private settings, and in compliance with the university’s protocols.

Given the sensitive nature of the topic, participants were informed about the availability of institutional health and counseling services should they experience any discomfort. A short debriefing was conducted after each interview to ensure participants’ well-being. Although no participant requested further support, these services were made accessible as part of the ethical safeguards. The researcher, also a faculty member, clarified their role as a colleague rather than an evaluator. This dual role is acknowledged as a potential limitation due to its possible influence on participant responses.

### Reflexivity

The author is a tenured faculty member at the study site and therefore occupies both insider (colleague) and researcher roles. The author also personally experiences DES, which may heighten sensitivity to participants’ symptom narratives and coping accounts. This proximity facilitated rapport and contextual understanding, but also posed risks of social desirability bias and confirmatory interpretation. To mitigate these risks, recruitment emails emphasized voluntariness, interviews were scheduled off-campus when requested, and a reflexive journal was maintained to document pre-existing assumptions (e.g., expectations that older faculty would report more severe strain) prior to and during analysis. In addition, two external qualitative scholars independently audited 20% of the transcripts to examine code-to-theme fit, quotation-to-theme alignment, and the consistency of analytic interpretations. Feedback from this audit informed minor refinements to theme boundaries and strengthened analytic rigor by ensuring that thematic interpretations remained grounded in participants’ accounts rather than researcher assumptions.

## Results

Descriptive phenomenological analysis of the nine individual interviews yielded four significant themes derived from multiple minor themes. The findings illustrate educators' complex and deeply personal experiences of coping with DES. These themes provided insights into the participants' perceptions, coping mechanisms, and institutional needs regarding DES. For a summary, see Table 1, Table 2.

**Table 1:** Interview Questions.

***Question Area***	***Main Questions***
***Screen Usage and Routine***	*Can you describe your daily routine involving digital screen use? How many hours do you typically spend on screens?*
***Experiences with DES***	*What symptoms of digital eye strain have you personally experienced? How do these symptoms impact your daily activities, both professionally and personally?*
***Coping Strategies and Institutional Support***	*What strategies or adjustments have you made to alleviate DES symptoms? How effective have these been? Do you think your institution provides adequate support for managing DES?*
***Impact on Professional Life and Ergonomics***	*Have your symptoms affected your productivity or job satisfaction? Have you made any ergonomic changes to your workspace, and have they helped?*
***Future Outlook and Suggestions***	*How do you see the role of digital technology evolving in education given the challenges of DES? What changes or improvements would you like to see?*

**Table 2:** Summary of Themes.

Major Themes	Minor Themes
Digital health and resilience	Recognizing symptom progression; Practicing immediate self-regulation and symptom management
Workstation and environmental ergonomics	Modifying workstations through improvised solutions; Managing lighting, glare, and workspace constraints; Seeking ergonomic assessment and resources
Work-life integration	Experiencing workload spillover and boundary erosion; Managing recovery constraints and daily functioning
Health services, policy supports, and system-level enablers	Noting the absence of eye-care provisions in health services; Strengthening institutional eEducation and wellness wupports; Normalizing reminders, break systems, and digital wellness practices; Calling for sector-level policy standards

To clarify thematic boundaries, each major theme was conceptualized as capturing a distinct layer of the DES experience. During analysis and revision, coded meaning units were re-examined to assess whether themes could be collapsed; however, participants’ accounts consistently reflected analytically separable dimensions. "Digital health and resilience" (Theme 1) reflects educators’ embodied awareness of DES symptoms and their immediate self-regulatory responses enacted in the moment (e.g., pacing work, eye drops, brief pauses, and symptom-triggered screen adjustments). "Workstation and environmental ergonomics" (Theme 2) centers on the physical conditions of screen work and ergonomic constraints within the immediate workspace (e.g., chair/table height, screen positioning, lighting/glare, and the need for ergonomic assessment/resources), rather than institutional health services or policy provisions. In Theme 1, brightness adjustment reflects in-the-moment symptom regulation; in Theme 2, it reflects workspace optimization to reduce environmental visual load. "Work-life integration" (Theme 3) captures temporal spillover and boundary erosion, describing how symptoms and digital workload extend work into rest periods and home life. "Health services, policy supports, and system-level enablers" (Theme 4) reflects organizational and sector-level supports (or their absence) that shape the sustainability of coping over time (e.g., eye-care provisions within health services, seminars/wellness programs, reminders or break systems that normalize recovery, and calls for CHED/DepEd standards). Quotations were assigned based on the primary analytic meaning they conveyed to maintain conceptual separation and minimize thematic overlap. To address concerns regarding quotation reuse, each verbatim excerpt was intentionally assigned to only one theme and was not repeated across thematic sections; assignment decisions were reviewed during analysis to ensure that illustrative examples were unique to each theme and aligned with its analytic focus. When an excerpt could plausibly fit more than one theme, it was retained under the theme that best captured its dominant analytic function.

### Theme 1: Digital health and resilience

This theme captures educators’ embodied awareness of DES symptoms and their immediate self-regulatory responses used to manage discomfort and sustain daily functioning. The meaning-units clustered here were symptom-triggered and reactive (bodily-cued coping), emphasizing moment-to-moment regulation rather than environmental or institutional change. Participants described a noticeable decline in visual well-being as their responsibilities became more computer-based. One educator shared, “I used to have no issues with my eyes, but now I can’t read words clearly” (P5). Another echoed progressive vision changes, stating, “I get annual eye check-ups, and every year my prescription increases” (P6), linking worsening eyesight to prolonged digital work.

Educators described classic DES symptoms—headaches, eye dryness or redness, stinging sensations, and blurred vision—that disrupted concentration and sometimes forced them to pause work. One participant emphasized the intensity of discomfort: “When I have been staring at the screen too long, my eyes feel hot and there’s numbness at the back of my head” (P5).

Despite these concerns, participants described personal coping practices enacted in response to bodily cues. These included the use of eye drops (“I use eye drops because I rarely get to rest from using gadgets” – P5) and self-initiated eye exercises (“I looked for eye exercises on YouTube to help with my eyestrain” – P5). Others attempted to break screen exposure through small interruptions such as meals or snacks: “I try to break up my screen time by eating or taking snack breaks” (P2).

Overall, the theme highlights a critical tension: although educators recognized preventive practices, sustained implementation was difficult under heavy digital demands and without consistent institutional reinforcement.

### Theme 2: Workstation & environmental ergonomics

This theme focuses on educators’ efforts to manage DES by modifying the physical workstation and environmental conditions of digital work (e.g., furniture, screen height, lighting, and glare). The meaning units clustered here were environmental and ergonomic, reflecting attempts to reduce visual and postural load through workspace optimization—often under constraints of limited resources or fixed classroom/office conditions. These accounts describe changes to the immediate work setting, rather than institutional health services or formal policy supports. Participants described improvised strategies to make their workstations more tolerable. One educator shared, “I even added a pillow to my chair just to be comfortable while using the laptop” (P8), while another described “doubling the chair to level it with the table” (P7), reflecting creative adjustments in the absence of ergonomic furniture.

Other strategies involved modifying visual conditions at the workstation. One educator noted, “Sometimes I adjust the screen brightness so it’s not too painful to the eyes” (P3). However, these adaptations were constrained by limited resources and suboptimal workspaces. One participant explained, “Even if the table isn’t balanced, I still keep working” (P6), suggesting a resigned acceptance of discomfort as part of routine work. Classroom conditions also contributed to strain: “In our classroom, sometimes the lighting hurts the eyes. It’s kind of dim” (P3).

Participants also emphasized the need for ergonomic assessment and workstation guidance to improve the physical conditions of screen-based work. As one faculty member stated, "There should be an ergonomic assessment for faculty" (P6), highlighting that discomfort was shaped not only by individual adjustment, but also by the availability of ergonomic resources.

### Theme 3: Work-life integration

This theme captures boundary erosion—how digital workload and DES symptoms spill over into home life and rest time, shaping work hours, recovery, and emotional strain. The meaning units in this theme were temporal and relational, highlighting delayed task completion, prolonged screen exposure, and reduced recovery that reshaped participants’ daily routines.

Participants described how screen-intensive work routinely extended beyond standard working hours. Several reported long daily exposure, such as, “When you put everything together, it reaches about eight to ten hours” (P1), and “All in all, my screen time is around eight to nine hours per day” (P3). Others described sustained after-hours workload, including, “There is a lot to do…. Every night, I spend as much as six to seven hours” (P2), and “In the evening, I still work starting around 9 p.m.” (P9). For some, exposure continued into home life: “I extend my work at home because sometimes tasks need to be finished” (P3), and “Even at home, I still bring my laptop and phone, so screen exposure never really ends” (P5).

Participants also described how DES symptoms slowed productivity and disrupted task completion, leading to further time spillover. One educator explained, “Sometimes I am unable to finish my work because of the symptoms” (P2), while another noted, “In my professional life, I expect to finish within eight hours, but I get delayed when I feel dizzy” (P6). Despite symptom burden, breaks were often limited: “My breaks are very minimal—really, only during lunch break” (P6). At the same time, some participants recognized how screen habits intruded into recovery periods, such as, “Using my phone before sleeping has become a habit, and I should reduce it” (P4).

Several accounts conveyed emotional and functional strain that went beyond physical discomfort. One participant shared that when headaches occur, “I pause during class because I start to feel irritable” (P5). Another described the intensity of prolonged exposure: “I almost vomited because of a migraine attack after long screen exposure” (P9). Others framed their experience as pushing through discomfort in order to meet work demands, stating, “You really have to push yourself to keep up with work” (P8), and “How can you function properly when you are not feeling well, especially with your eyes?” (P8). In response, some educators attempted to protect recovery time through boundary-setting strategies, such as, “Every Sunday, I limit myself from working and avoid opening my laptop” (P9), while others explicitly emphasized the need for balance: “Work–life balance is really necessary when it comes to using digital screens” (P2).

Taken together, these accounts indicate that educators experienced DES not merely as physical discomfort, but as a fatigue-inducing and recovery-limiting work condition. Participants consistently described pushing through eye pain, headaches, and visual discomfort to meet academic demands, often resulting in prolonged screen exposure and minimal opportunities for rest. Expressions such as “My breaks are very minimal” (P6), “You really have to push yourself to keep up with work” (P8), and “Screen exposure never really ends” (P5) reflect sustained exhaustion and difficulty disengaging from digital work. Across accounts, educators described sustained exhaustion and reduced recovery as they “pushed through” discomfort with minimal breaks, suggesting that DES combined with digitally intensified workloads contributed to chronic fatigue and strain that resemble burnout-related processes.

### Theme 4: Health services, policy supports, and system-level enablers

Unlike Theme 2, which focuses on the physical workstation environment, this theme emphasizes system-level supports—health services, formal policies, reminders, and institutional accountability—that enable (or limit) sustained coping. The meaning units clustered here were systemic and resource-oriented, centered on whether the university (and the wider sector) provides structures that normalize eye health practices beyond individual self-management. Participants described gaps in routine health provisions (e.g., annual exams), the absence of organized digital wellness supports, and calls for institution-wide or sector-level standards.

Participants consistently emphasized the absence of structured institutional support for digital eye health. One participant stated, “There is no support system for digital eye strain” (P4), while another noted, “There’s no support provided by a public state university in the Philippines for digital eye strain” (P7). Participants further highlighted that eye care was not integrated into routine workplace health provisions, with remarks such as, “Eye care is not included” (P4), and “There’s no eye check-up included in the annual physical exam” (P5).

When participants spoke about eye examinations, they did not describe these as tools to diagnose DES itself. Rather, participants framed eye check-ups as preventive and monitoring measures intended to track vision changes associated with prolonged screen exposure, as reflected in statements such as, “I hope there will be an eye check-up included in the annual physical exam for faculty” (P5), and “It would really help if eye check-ups were included in the annual physical exam” (P4).

Participants’ “hope” statements thus reflected aspirational requests for preventive services rather than contradictions of earlier reports of absence. These statements expressed a desire for institutional action, including routine eye examinations, access to basic eye care resources, and educational initiatives. One participant shared, “I hope the university provides this kind of health service, even just a simple seminar about the eyes” (P4).

Technological supports were also viewed as potentially helpful in reinforcing protective routines. One educator remarked, “I wish there were reminders or breaks to lessen the burden on the eyes” (P8), suggesting openness to system-level tools that normalize recovery. Beyond the institution, participants also called for broader policy support, noting, “I hope that the Department of Education or CHED will also implement policies regarding this” (P5), highlighting the perceived value of sector-wide standards to strengthen institutional accountability for digital eye health.

## Discussion

This study explored the lived experiences of university educators dealing with DES in an increasingly digital teaching landscape. The findings provide a nuanced understanding of how DES affects educators not only physically, but also in terms of productivity, emotional well-being, and coping behavior, highlighting the interplay between individual self-regulation, workplace conditions, and institutional support.

Participants reported common DES symptoms, including eye fatigue, blurred vision, dryness, headaches, and musculoskeletal discomfort, consistent with prior occupational health research (Blehm et al., 2005, Munshi et al., 2017). Although these symptoms were often normalized within academic work cultures, they were described as disruptive to teaching performance, assessment tasks, and sustained concentration. Several participants expressed concern about progressive vision changes, reinforcing arguments by Meneses-Claudio et al. (2023) that DES among educators should be treated as a public health concern with implications for instructional quality and educational sustainability.

Beyond physical discomfort, the findings indicate that DES is embedded within a broader psychosocial context shaped by technostress and burnout-relevant strain processes. While participants did not explicitly label their experiences as “burnout,” the findings demonstrate a clear data-based pathway linking DES to exhaustion, cognitive overload, and reduced recovery. As shown in Theme 3 (Work–Life Integration), educators described how DES symptoms slowed task completion and extended screen exposure into evenings and home life, such as “Sometimes I am unable to finish my work because of the symptoms” (P2) and “In the evening, I still work starting around 9 p.m.” (P9). This pattern is consistent with evidence that nighttime technology use can disrupt sleep quality and recovery, potentially intensifying fatigue and psychological strain among digitally engaged professionals (Ahmed et al., 2025).

Importantly, participants’ accounts also reflected emotional and functional strain, not only physical discomfort. One educator described pausing work because headaches led to irritability, stating, “I pause during class because I start to feel irritable” (P5). And as noted, another described severe overload following prolonged screen exposure, mentioning that they nearly vomited in connection with a migraine attack after long screen exposure (P9). Others framed their experience as pushing through discomfort to meet work demands, explaining, “You really have to push yourself to keep up with work” (P8). Together, these accounts indicate that DES functioned as a compounding occupational stressor, amplifying technostress by reducing efficiency, extending work hours, and limiting psychological disengagement from digital work. This tension reflects broader technostress scholarship noting that digital technologies may produce both strain and potential techno-eustress depending on demands, resources, and organizational supports (Nascimento et al., 2024).

These experiences align with Tarafdar et al.’s (2007) concepts of techno-overload and techno-invasion, wherein persistent digital demands blur work–life boundaries and intensify occupational strain. In this study, DES did not operate in isolation; rather, it interacted with digitally intensified workloads to exacerbate exhaustion and reduce recovery, thereby increasing vulnerability to burnout-related outcomes (Hidayati et al., 2025). This linkage is not inferred solely at a conceptual level, but is grounded in participants’ descriptions of delayed task completion, irritability, physical overload, and difficulty disengaging from screen-based work.

The distinction between resilience and adaptation further clarifies how educators responded to these challenges. Resilience, as reflected in the findings, referred to individual-level, self-regulatory coping behaviors, such as adjusting screen brightness, pacing work, using eye drops, or taking short breaks in response to bodily cues. These strategies demonstrated educators’ awareness of symptom triggers and their efforts to maintain functioning despite discomfort. In contrast, adaptations pertained to environmental or structural modifications, including workspace adjustments, ergonomic changes, or institutional provisions that altered the conditions under which digital labor occurred. The findings show that while educators frequently exercised personal resilience, their capacity to sustain healthy practices was constrained in the absence of supportive adaptations at the organizational level.

Although many participants attempted to apply ergonomic recommendations—such as modifying screen settings or taking breaks—these practices were inconsistently implemented. High workloads, performance pressures, and expectations of constant availability often undermined sustained self-care. This supports The Vision Council’s (2016) position that individual behavior change alone is insufficient without enabling organizational systems. In this context, resilience without institutional reinforcement risks becoming a form of endurance rather than effective coping.

The study also highlights persistent gaps between national occupational health policies and institutional practice in the Philippine context. While Republic Act No. 11058 (Occupational Safety and Health Law), Civil Service Commission guidelines (CSC, 1992), and Department of Labor and Employment (DOLE) break-time standards provide a regulatory foundation for worker wellness (Respicio & Co., 2025), participants reported limited translation of these policies into concrete university-level initiatives. The absence of ergonomic assessments, structured screen breaks, and formal digital wellness programs reflects broader implementation challenges, including competing institutional priorities, limited monitoring mechanisms, and a productivity-oriented culture that marginalizes faculty well-being.

Importantly, participants did not view vision examinations as diagnostic tools for DES itself. Rather, eye check-ups were perceived as preventive and monitoring mechanisms that could help detect vision changes associated with prolonged screen exposure and guide appropriate corrective or ergonomic interventions. This distinction underscores the need to frame institutional eye care provisions as part of comprehensive occupational health strategies rather than as standalone clinical solutions.

These findings are well explained through the transactional theory of stress and soping (Lazarus & Folkman, 1984). Educators appraised DES as a threat to their professional functioning (primary appraisal) and mobilized coping responses that were either problem-focused (e.g., workspace adjustments) or emotion-focused (e.g., enduring discomfort, pushing through work demands). When coping resources were unsupported by institutional structures, participants described fatigue, irritability, and reduced control—conditions that heighten susceptibility to burnout. Strengthening organizational support thus becomes critical not only for symptom reduction, but also for sustaining adaptive coping capacity.

**Fig. 1:**
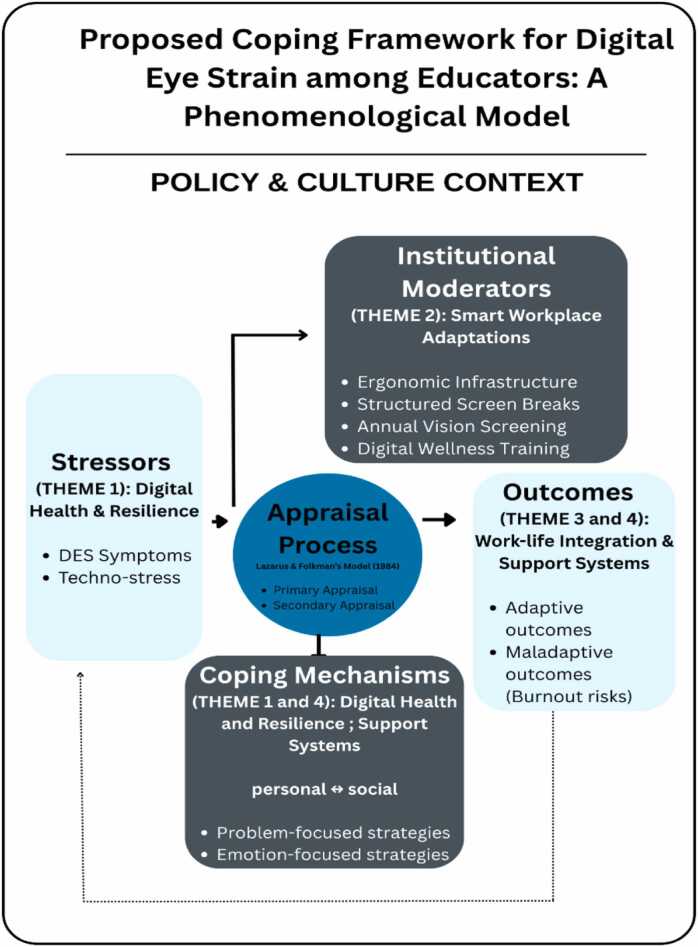
Proposed coping framework for Digital Eye Strain among educators. Source: Author’s own work.

Fig. 1 presents a proposed coping framework illustrating how DES-related stressors interact with technostress, individual appraisal processes, and coping strategies within institutional and policy contexts. The framework demonstrates how outcomes diverge toward resilience or continued strain depending on the availability of ergonomic resources, supportive policies, and cultural norms surrounding digital work. This model situates DES as both a physical and psychosocial occupational issue shaped by multi-level influences.

Finally, the findings align with international literature while offering context-specific insight. Studies from Turkey (Liman Kaban & Kaynar Zehir, 2023) and India (Shet Parkar et al., 2024) similarly report digital exhaustion among educators, though often linked to limited awareness. In contrast, participants in the present study demonstrated awareness, but remained constrained by structural inaction. This suggests that the central challenge lies not in knowledge deficits, but in the absence of institutional mechanisms that translate awareness into sustainable practice. Addressing DES among educators therefore requires a shift from individual responsibility toward integrated organizational and policy-level interventions.

### Implications

The implications of this study span the practical, institutional, and policy domains. At the institutional level, academic workplaces must move beyond encouraging individual behavioral change and instead create supportive ecosystems that reduce digital fatigue and enhance educator well-being. Guided by Lazarus and Folkman’s (1984) transactional theory of stress and coping, educators perceive DES as a chronic stressor that demands adaptive coping resources. To enhance their coping capacity, institutions must embed both preventive and responsive measures into their systems.

The foremost among these is the need for regular ergonomic assessments. As participants recounted, many relied on makeshift workstation adjustments, doubling chairs, using pillows, or enduring poor lighting due to the absence of appropriate furniture or technical support. The provision of adjustable chairs, anti-glare screens, external keyboards, and monitor risers can significantly reduce visual and musculoskeletal strain (Gushgari et al., 2024, The Vision Council, 2016). These interventions serve as problem-focused coping tools that directly target the environmental contributors to DES.

Moreover, structured screen break policies should be implemented and monitored. For example, scheduling a 10-minute rest every two hours of continuous screen use, in line with DOLE recommendations (Respicio & Co., 2025), and institutionalizing the 20–20–20 rule can provide much-needed relief to digital workers. Such policies, though simple, can have measurable effects on both symptom reduction and mental clarity.

Faculty development initiatives should integrate digital ergonomics and ocular health training as a core component of educator wellness. These training sessions must cover essential topics such as proper posture, optimal screen positioning, appropriate lighting, and preventive eye exercises (Bayındır & Gültepe, 2022). Moreover, university health services should be expanded to include annual vision screenings, partnerships with optical clinics, subsidies for protective eyewear, and the implementation of corporate-style wellness programs tailored to academic environments. Structured training models have already demonstrated success in other educational contexts such as a school-based eye health education program that significantly improved knowledge and practices among students (Park & Ahn, 2022) suggesting that concise, skill-based curricula can be effectively adapted for university faculty as well.

It is equally important to cultivate a digital self-care culture. Drawing from Tarafdar et al.’s (2007) technostress model, which identifies techno-overload and techno-invasion as key stressors, administrators must discourage after-hours communication, avoid consecutive virtual meetings, and normalize recovery periods. Gabbiadini et al. (2023) emphasized that these forms of institutional support reduce technostress and help educators maintain well-being and professional satisfaction. Embedding such practices into institutional culture not only protects health, but also boosts job satisfaction and retention.

Beyond practical well-being, DES has implications for instructional quality. As this study suggests, educators experiencing discomfort often reduce interaction, shorten lectures, and avoid cognitively demanding tasks, thereby diminishing the richness of digital pedagogy (Marrinhas et al., 2023, Wang et al., 2024). In this light, protecting educators’ vision and energy is essential for preserving the effectiveness and equity of hybrid and online education.

At the policy level, regulatory bodies, such as the Commission on Higher Education (CHED) and the Civil Service Commission (CSC), are positioned to lead systemic change. CHED, for instance, could issue memoranda-mandating DES prevention measures in state universities, including workload pacing and screen-time limits, aligned with national laws such as Republic Act No. 11058 (Republic of the Philippines, 2018) and CSC wellness directives (1992). Policies that draw from international innovations such as the “Right to Disconnect” law can also be adapted to the Philippine context to help educators regain control over their digital time.

In parallel, faculty unions and academic labor organizations can leverage these findings to advocate for greater recognition of DES as a work-related condition warranting institutional accommodation. Through collaborative advocacy, policies that remain theoretical can be transformed into everyday protection.

## Conclusion

The findings of this study illuminate the complex and multifaceted experiences of university educators managing DES within an increasingly digital teaching environment. Through a descriptive phenomenological lens, it became evident that prolonged screen exposure, compounded by inadequate ergonomic support and insufficient rest periods, adversely affects not only educators’ ocular health, but also their overall well-being and job performance. Participants described a range of symptoms, including eye fatigue, dryness, headaches, and visual disturbances, which disrupted their ability to deliver instruction, engage with students, and manage academic tasks effectively. These accounts are consistent with earlier literature that identifies DES as a prevalent occupational health issue among screen-dependent professionals (Blehm et al., 2005, Meneses-Claudio et al., 2023, Munshi et al., 2017).

Although many participants developed coping strategies, such as screen brightness adjustment, use of eye drops, or self-imposed breaks, these practices were inconsistently applied, largely due to workload demands and the absence of institutional reinforcement. The tension between individual knowledge and institutional neglect is palpable throughout the narratives. Despite national wellness policies such as the Republic Act No. 11058 (Republic of the Philippines, 2018), CSC circulars (1992), and DOLE’s visual terminal use guidelines (Respicio & Co., 2025), educators reported a lack of structured support systems, revealing a disjuncture between policy mandates and everyday workplace realities.

Taken together, the study underscores that DES is not merely a personal health inconvenience, but a systemic challenge that, if left unaddressed, may contribute to educator fatigue, burnout, and a decline in instructional quality. However, the hopeful aspect of these findings lies in the solutions educators themselves proposed, ranging from regular eye check-ups and ergonomic upgrades to policy-driven screen-break routines. These insights affirm the urgent need for academic institutions to prioritize faculty wellness as a strategic imperative, not only for occupational health, but also for sustaining high-quality digital learning.

## Limitations and future research

Although this study offers valuable insights, it has several limitations that must be acknowledged. First, the small and localized sample of nine faculty members from a single public university limits generalizability. Future research could include participants from private institutions, rural colleges, and satellite campuses for a broader comparison. Second, self-selection bias may have occurred, as those who were more aware of our concern about DES may have been more likely to participate. This could mean that those most severely affected or unaware of their symptoms were underrepresented. Third, the researcher’s dual role as faculty member and interviewer may have influenced responses or introduced subtle bias despite efforts to mitigate this through bracketing and reflexive journaling. Fourth, the study focused only on higher education faculty, excluding K–12 educators who may experience DES differently due to varied screen routines and institutional conditions (Shet Parkar et al., 2024). Fifth, while general screen use was explored, specific digital tools (e.g., Zoom, LMS, and smartphones) were not analyzed, which could be examined in relation to DES severity (The Vision Council, 2016). Lastly, institutional perspectives, such as those from administrators, IT, or human resources staff, were not included. Future studies could adopt mixed methods designs, integrate stakeholder inputs, and explore action research or longitudinal approaches to assess the effectiveness of wellness interventions.

## Author statement

The researcher was responsible for the conceptualization, methodology, and investigation of the study. Data collection, analysis, and interpretation were conducted by the researcher, who also drafted the original manuscript. The researcher also contributed to the writing, review, and revision of the final manuscript. The researcher has read and approved the final version of the manuscript.

## Declaration of Generative AI and AI-assisted technologies in the writing process

The author declares that artificial intelligence (AI)–assisted tools were used solely for language refinement, clarity, and editorial support during manuscript preparation. AI tools were not used for data collection, data analysis, interpretation of findings, or generation of results. All substantive content, methodological decisions, interpretations, and conclusions remain the sole responsibility of the author.

## Ethics Approval

This study was conducted in accordance with the ethical principles outlined in the Declaration of Helsinki and was approved by the university’s Institutional Research Ethics Committee. Prior to participation, all participants received detailed information about the study's purpose, procedures, and their rights, and provided written informed consent.

## Funding

The researcher confirmed that no external funding, financial sponsorship, or institutional grants were received for this study. This research was conducted independently, with no financial support influencing the study design, data collection, analysis, or manuscript preparation.

## Declaration of Competing Interests

The authors declare that they have no known competing financial interests or personal relationships that could have appeared to influence the work reported in this manuscript.
